# Off-Season Training Habits and BMI, Not Preseason Jump Measures, Are Associated with Time-Loss Injury in Female Collegiate Soccer Players

**DOI:** 10.3390/sports8030036

**Published:** 2020-03-15

**Authors:** Jason Brumitt, Alma Mattocks, Amy Engilis, Jill Sikkema, Jeremy Loew

**Affiliations:** 1 School of Physical Therapy, George Fox University, 414 N. Meridian St., Newberg, OR 97132, USA; 2 School of Kinesiology, Counseling, and Rehabilitative Sciences, Northern Kentucky University, Highland Heights, KY 41099, USA; mattocksa1@nku.edu; 3 Division of Natural Sciences and Health, Warner Pacific University, Portland, OR 97215, USA; aengilis@warnerpacific.edu; 4 Sports Medicine Department, George Fox University, Newberg, OR 97132, USA; jsikkema@georgefox.edu; 5 Sports Medicine Department, Lewis and Clark College, Portland, OR 97219, USA; jloew@lclark.edu

**Keywords:** epidemiology, football, functional performance test, injury screening, preseason

## Abstract

The primary purpose of this study was to determine the effectiveness of the standing long jump (SLJ) and the single-leg hop (SLH) tests to discriminate lower quadrant (low back and lower extremities) injury occurrence in female collegiate soccer players. The secondary purpose of this study was to determine associations between injury and off-season training habits or anthropometric measures. SLJ, SLH, and anthropometric measures were collected during a preseason screening clinic. Each subject completed a questionnaire providing demographic information and off-season training habits. Each athlete performed three SLJ and three SLH per leg. SLJ and SLH scores were not associated with an increased risk of a noncontact time-loss lower quadrant (LQ) injury. Athletes with a higher BMI or who reported less time training during the off-season were two times more likely to sustain an injury. Athletes who had both a higher body mass index (BMI) and lower off-season training habits were three times (relative risk = 3.1 (95% CI: 1.7, 5.5) *p*-value = 0.0001) more likely to sustain a noncontact time-loss lower quadrant injury. Preseason SLJ and SLH scores do not discriminate injury risk in female collegiate soccer players. Higher BMI and lower off-season training habits are associated with an increased risk of LQ injury.

## 1. Introduction

Soccer has one of the highest time-loss injury rates (range 7.7 to 8.07 per 1000 athletic exposures) of all sports played by female athletes at the collegiate level in the United States [1,2]. Over 70% of all soccer-related musculoskeletal injuries that occur at the collegiate level involve the lower extremities (67.8% of all injuries during games and 72% of all injuries during practices) and the trunk/back region (6.3% of all injuries during games and 4.2% of all injuries during practices) [3]. The most common sport-related injuries (excluding concussions) experienced by this population of athletes include ligament sprains of the ankle, internal derangement of the knee (e.g., cruciate or collateral ligament sprain, meniscal injury), and muscle-tendon strains of the thigh [3].

Sports medicine professionals and coaches often administer functional performance tests (FPTs) during the off-season or preseason to identify athletes who may be at risk for injury [4,5]. An FPT is an assessment tool designed to serve as a clinical correlate for a laboratory test (e.g., single-leg hop tests may be used to evaluate progress during rehabilitation if isokinetic testing is unavailable) [6,7]. Additional advantages of FPTs are that they are inexpensive to administer and do not require special training for the tester [5]. Two functional performance tests, the standing long jump (SLJ) and the single-leg hop (SLH) for distance, have shown promise as tools to discriminate injury risk in female collegiate athletes [8,9,10]. A heterogeneous population (i.e., athletes representing eight sports including soccer) of National Collegiate Athletic Association (NCAA) Division III female athletes who had a greater than 10% difference in SLH measures between limbs were four times more likely to experience a noncontact time-loss foot or ankle injury [8]. NCAA Division III female collegiate athletes (a heterogeneous sample including soccer players) were nine times more likely to experience a noncontact time-loss thigh or knee injury if one started the season with an SLJ <80% of one’s height, bilateral SLH measures <65% of one’s height, and a lower extremity functional test (an agility drill) of 118 seconds or more [9]. A homogeneous sample of female collegiate volleyball players who had lower SLJ (<80% height) and SLH (<70% height and a >10% side-to-side asymmetry) scores were four times more likely to experience a time-loss injury to the low back or lower extremity during the season [10]. Lower preseason SLJ and SLH scores have served as a marker for injury in heterogeneous populations and volleyball athletes; however, it is unknown if these tests can discriminate injury occurrence in a homogeneous population of female collegiate soccer athletes.

Many female soccer players return to campus for preseason training each August after having been off-campus for the summer break. Athletes are typically provided with an off-season training program; however, individual adherence is not directly supervised. Not adequately training during the off-season may also increase one’s risk of injury. For example, male collegiate soccer players who devoted less time to strength and conditioning training during the off-season period prior to the start of the preseason were three times more likely to experience a noncontact time-loss injury to the low back or lower extremity [11]. It is currently unknown if off-season training habits increase the risk of injury in female collegiate level soccer players.

Another potential risk factor for musculoskeletal injury that warrants assessment in female collegiate soccer players is body mass index (BMI). Several studies have identified BMI as a risk factor for injury in soccer players [12,13,14,15,16]. A greater BMI was associated with a 1.43 times greater risk of injury in teenage female soccer players and a 1.51 times greater risk of lower extremity (LE) injury in elite female soccer players [13,14]. Athletes with a higher BMI may lack the ability to stabilize the ankle during sport-specific movements (i.e., cutting, changing momentum) [15].

The primary outcome of this study was to determine if the SLJ or the SLH tests could be used as preseason screening tools to identify female collegiate soccer players at risk for a sport-related injury to the lower quadrant region (i.e., low back and lower extremities) region or to a specific region of the lower quadrant (e.g., thigh and knee region). It was hypothesized that shorter preseason jump and hop scores would be associated with a greater risk of a noncontact time-loss sports injury to the lower quadrant or to a specific region of the lower quadrant. The secondary outcome of this study was to determine if associations exist between injury and off-season training habits or anthropometric measures. It was hypothesized that a greater BMI or lower off-season training volumes would be associated with a greater risk of noncontact time-loss lower quadrant injuries.

## 2. Methods

### 2.1. Participants

A total of 119 female collegiate soccer players (mean age 19.2 years ± 1.2; mean height 1.65 m ± 0.06; mean weight 62.6 kg ± 8.6; and mean BMI 22.9 kg/m^2^ ± 2.6), representing athletes from the NCAA Division III (5 teams; n = 102) and National Association of Intercollegiate Athletics (NAIA) (1 team; n = 17) levels of competition, were studied. Recruitment of soccer players occurred in a two-step process. First, the primary investigator (PI) contacted the team’s head coach and athletic trainer (ATC) via phone or email to recruit team participation. If the head coach and ATC agreed to allow their team to be tested, the PI recruited athlete participation via email. An athlete was excluded from study participation if she was under the age of 18 years. The Institutional Review Board of George Fox University (Newberg, OR, USA) approved this study. Athletes provided written consent prior to study participation.

### 2.2. Testing Protocol

The following data were collected from each athlete at the start of the preseason: demographic information, height, weight, and functional performance test measures. First, each athlete completed a questionnaire collecting the following information: age and the number of hours per week, for the six week period prior to the start of the preseason, devoted to the training categories of weightlifting, cardiovascular exercise (e.g., running), plyometric exercise, and scrimmaging. Next, height (using a cloth measuring tape affixed to the edge of wall) and weight (using a standard medical scale) were collected. Athletes next performed a five-minute dynamic warm-up consisting of the following activities: forward lunging, backward lunging, walking on heels, walking on toes, and high knee marching [11]. After completing the dynamic warm-up, each athlete completed six SLJ (three performed at 50% effort and three performed at 100% effort) and three SLH per each lower extremity. Functional performance test measures were collected by one investigator (PI).

As aforementioned, the SLJ was performed first. The athlete was instructed to stand with her feet positioned shoulder-width apart behind the starting line (i.e., a piece of athletic tape applied to the floor). A cloth measuring tape (unit of measurement in inches; converted to meters for statistical analysis), oriented perpendicular to the starting line and affixed to the floor with tape, was used for measuring the distance jumped. After performing the three submaximal effort jumps, athletes were instructed to perform three maximal effort jumps. Athletes were required to perform the SLJ with hands clasped behind the back [17]. An SLJ was scored if the athlete was able to land under control holding the landing for five seconds [17]. If the athlete failed to land under control, that trial was repeated. The mean of the three SLJ trials, normalized to height (mean SLJ score/ height), was used for data analyses. The primary investigator previously reported test–retest reliability (ICC_3,3_) for the SLJ (0.96 (95% confidence interval: 0.83–0.97)) [18].

The SLH trials were performed after completing the SLJ trials. The SLH trials alternated between lower extremities with a coin toss determining which lower extremity was hopped off first. Each SLH trial was performed with the athlete clasping her hands behind her back [17]. A trial was repeated if the athlete was unable to land under control or if she was unable to hold the landing for 5 seconds [17]. The mean of the three hop trials, per side, normalized to height (mean SLH score/height), was used for data analyses. The PI previously reported test–retest reliability (ICC_3,3_) for the (R) SLH (0.95 (95% confidence interval 0.89–0.98)) and (L) SLH (0.96 (95% confidence interval 0.89–0.98)) [18].

### 2.3. Injury Surveillance

Each team’s ATC collected injury information during the course of the approximately four-month season. The following data were collected per each time-loss injury: injury mechanism (only noncontact injuries were used for data analyses), region of the body, and injury diagnosis. The operational definition of an injury was any muscle, joint, or bone problem/injury of the lower back or the lower extremity (categorized by the following regions: hip, thigh, knee, leg, ankle, or foot) that occurred during practice or a game that resulted in the athlete either having been removed from that day’s event or requiring the athlete to miss a subsequent sanctioned event [8,19]. The PI collected injury records on a weekly basis from each team’s athletic trainer.

### 2.4. Statistical Analysis

An a priori sample size was calculated indicating a minimum recruitment goal of 102 subjects. This calculation was based on an estimation of 25% of at-risk athletes experiencing an injury, an alpha level set at 0.05, and a power of 0.80. A total of 119 female collegiate soccer players were recruited for this study.

Descriptive statistics (mean ± standard deviation (SD)) were calculated for age, anthropometric measures, off-season training habits, and functional performance test measures. Mean functional performance test scores were normalized as a percentage of body height (mean functional performance test /height). Asymmetry between limbs during the SLH was calculated by comparing the difference between one’s right and left SLH. Independent t-tests were used to compare means between athletes for age, anthropometric measures, training habits, and functional performance test scores based on injuries during the season.

**Figure 1:**
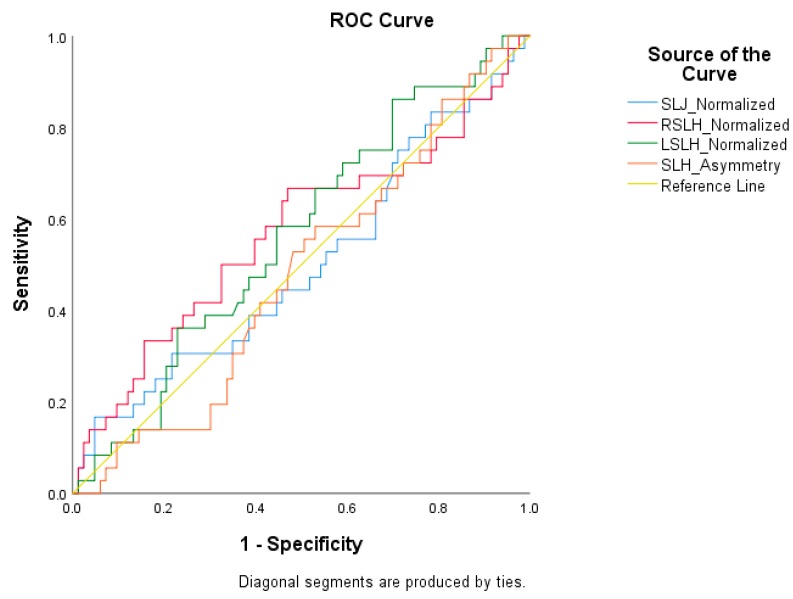
Receiver operator characteristic curve for functional performance tests and injury. SLJ normalized = standing long jump normalized to height; RSLH normalized = right single-leg hop normalized to height; LSLH normalized = left single-leg hop normalized to height; SLH asymmetry = side-to-side difference between SLH tests.

**Figure 2:**
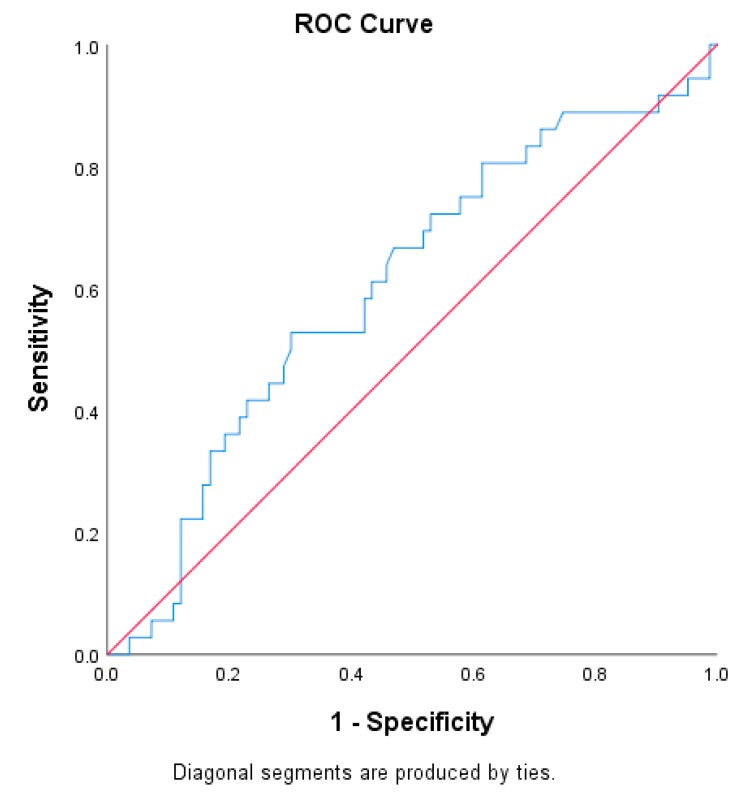
Receiver operator characteristic curve for body mass index and injury.

**Figure 3:**
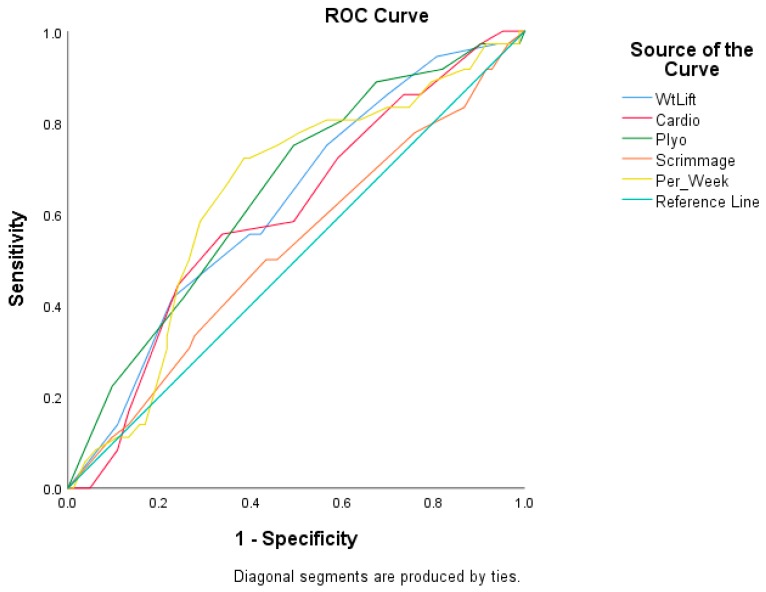
Receiver operator characteristic curve for off-season training habits during the six-week period prior to the start of the season and injury. WtLift = weightlifting; Cardio = cardiovascular exercise; Plyo = plyometric exercise; Per Week = total time devote to training per week.

Receiver operator characteristic (ROC) curves were constructed to identify cutoff scores for anthropometric measures, off-season training habits, and functional performance tests to dichotomize athletes into at risk and reference groups (Figure 1, Figure 2 and Figure 3). ROC curves failed to identify a cutoff score that maximized sensitivity and specificity per each functional performance test; therefore, previously reported cutoff scores were used to dichotomize athletes (Figure 1) [17]. The cutoff score for the SLJ was 79% of one’s height or less [at risk]/≥80%. The cutoff score for the SLH was 69% of one’s height or less (at risk)/≥70% (reference). The cutoff score for the limb asymmetry during the SLH was greater than 10% (at risk)/≤10% (reference). Risk profiles based on suboptimal performance on a battery of functional performance tests were also analyzed [9,10]. The first risk profile based on a battery of tests was dichotomized by the following: athletes who had an SLJ 79% of one’s height or less and each SLH 69% of one’s height or less [at risk]/all other athletes [10]. The second risk profile based on a battery of tests was dichotomized by the following: athletes who had an SLJ 70% of one’s height or less, each SLH 69% of one’s height or less, and a side-to-side asymmetry between SLH scores >10% (at risk)/all other athletes [9,10].

ROC curves for BMI (Figure 2) and for some of the off-season training habits (weightlifting, cardiovascular exercise, plyometric exercises, and total training hours per week; Figure 3) helped to identify cutoff scores. The cutoff score for BMI was 21.5 kg/m^2^ or less (reference)/greater than 21.5 kg/m^2^ (at risk) (area under curve 0.603 (95% CI: 0.492, 0.714)). The cutoff score for off-season weightlifting habits was 4 h or more per week (reference)/less than 4 h per week (at risk) (area under curve 0.621 (95% CI: 0.515, 0.728)). The cutoff score for cardiovascular exercise was 5 h or more per week (reference)/less than 5 h per week (at risk) (area under curve 0.602 (95% CI: 0.493, 0.711)). The cutoff score for off-season plyometric exercise habits was 3 h or more per week (reference)/less than 3 h per week (at risk) (area under curve 0.651 (95% CI: 0.545, 0.756)). The cutoff score for total training time per week was 14.75 or more hours per week (reference)/less than 14.75 h per week (at risk) (area under curve 0.641 (95% CI: 0.533, 0.749]). The ROC curve for scrimmaging did not identify a cutoff score; therefore, the mean score associated with scrimmaging was used to dichotomized athletes into an at risk group (i.e., less time devoted to scrimmaging) and a reference group (i.e., more time devoted to scrimmaging).

Cutoff scores were used to calculate the relative risk (RR) of a noncontact time-loss lower quadrant injury and noncontact time-loss injury to the thigh and knee region based on individual functional performance tests scores, performance on a battery of functional performance tests, anthropometric measures, off-season training habits, or a combination of demographic factors and functional performance test scores. Relative risk was not calculated for other regions of the lower quadrant (e.g., foot and ankle region) due to a lack of injuries in those areas. Data analyses were performed using SPSS Statistics 24 (Chicago, IL, USA) with the alpha level set at 0.05. 

## 3. Results

Mean (±SD) age, anthropometric, off-season training habits, and functional performance test scores are presented in Table 1. Thirty-six athletes (30.3% of the population) experienced a noncontact time-loss lower quadrant injury during the study. Injured athletes reported significantly less time training in each off-season exercise category except for cardiovascular exercises and scrimmaging. Injured athletes also reported less total training time per week during the six-week period prior to the start of the preseason when compared with their uninjured counterparts. There was no difference in functional performance test scores between groups.

Relative risk of a noncontact time-loss injury based on potential risk factors is presented in Table 2 and Table 3. There was no association between individual functional performance test measures or asymmetry during SLH and injury (Table 2). There was also no association between injury and performance on a battery of functional tests. 

Significant associations between anthropometric measures and/or off-season training habits and time-loss injury were found (Table 3). Athletes with a BMI greater than 21.5 kg/m^2^ were two times more likely (RR = 2.0 (95% CI: 1.0, 4.2) *p*-value = 0.04) to experience a lower quadrant injury during the season. The sensitivity (Sn) and specificity (Sp) associated with BMI as a risk factor for injury was Sn = 80.6 (95% CI: 64.0, 91.2) and Sp = 38.6 (95% CI: 28.1, 49.9). Two training categories were associated with a greater risk of lower quadrant (LQ) injury. Athletes who performed less than 3 h of plyometric exercises per week or who performed less than 14.75 h per week of training were two times more likely to experience a noncontact time-loss lower quadrant injury (Table 3). Athletes with a higher BMI and who reported less time devoted to off-season training were also more likely to be injured. Athletes were three times (RR = 3.1 (95% CI: 1.7, 5.5) *p*-value = 0.0001) more likely to experience a noncontact time-loss lower quadrant injury and two times (RR = 2.8 (95% CI: 1.2, 6.6) *p*-value = 0.014) more likely to experience a noncontact time-loss injury to the thigh and knee region if one’s BMI was greater than 21.5 kg/m^2^ and if one performed less than 14.75 h of total training per week.

## 4. Discussion

Previous studies have reported associations between preseason SLJ and/or SLH performance and injury in heterogeneous populations of female collegiate athletes [8,9]. Studies evaluating the ability of functional performance tests to discriminate injury risk in homogeneous populations are warranted because there can be differences in risk profiles based on sample characteristics (i.e., heterogeneous versus homogeneous samples) [20,21]. This study found that female collegiate soccer players (i.e., a homogeneous population) with shorter jump and hop distances were no more likely to experience a noncontact time-loss lower quadrant injury than their counterparts. There were, however, associations between injury and BMI and/or off-season training habits. These findings may help clinicians and coaches identify athletes at risk for injury.

Identifying athletes at risk for injury at the start of the season would be of benefit to both the athlete and the team. If an athlete could be identified as “at risk” a training program could be implemented to address deficits. Functional performance tests used as part of a preseason screening clinic could potentially provide coaches and sports medicine staffs with means to quickly and inexpensively screen athletes [4,8,9,10,11,20,21]. Prior studies have reported associations between preseason functional test scores and subsequent injury in a heterogeneous population of Division III female collegiate athletes [8,9]. Female athletes with a greater than 10% difference between limbs during the SLH test were four times more likely to experience a noncontact time-loss foot or ankle injury [8]. Female athletes were nine times more likely to experience a noncontact time-loss injury to the thigh and knee region if their SLJ was <80% height, bilateral SLH scores were <65% height, and their lower extremity functional test ≥118 seconds [9]. It is possible that this current study found no relationship between test scores and injury because this homogeneous sample (i.e., soccer players) was not influenced by the risk profiles from athletes representing other sports [8,9]. It is also possible that the initial associations between injury and functional test performance cannot be validated in subsequent cohorts. Initial reports of associations between injury and preseason scores on the Y-Balance Test, the Functional Movement Screen, and the lower extremity functional test have not been validated in subsequent studies [8,10,21,22,23,24,25,26]. Although there were no significant findings associated with functional performance test scores and injury in this study, the normalized SLJ and SLH data presented may be of use to sports medicine professionals when evaluating an athlete’s ability to return to sport after an injury [17].

BMI and off-season training habits were associated with injury in this study. It was hypothesized that a higher BMI would be associated with a greater risk of injury. In this study, a higher BMI was associated with a two-fold increased risk of a noncontact time-loss lower quadrant injury. It is important to appreciate, though, that a “higher” BMI did not necessarily mean that one was “overweight” or “obese”. The mean BMI for the injured athletes (n = 36) in this study was 23.3 (± 2.4) kg/m^2^ (a “healthy weight” BMI range is 18.5 to 24.9 kg/m^2^). Of the 36 injured athletes, only 25.0% were not in the “healthy weight” category: 7 athletes were in the overweight category (BMI range 25 to 30 kg/m^2^), 1 athlete was in the obese category (BMI 30 kg/m^2^ or more), and one athlete was in the underweight category (BMI less than 18.5 kg/m^2^). This finding of a higher BMI being associated with a greater risk of injury is consistent with prior studies [13,14,16]. A higher BMI (>21.5 kg/m^2^) and lower total training time per week (<14.75 h/w) was also associated with a greater risk of LQ injury (RR = 3.1 (95% CI: 1.7, 5.5)). An athlete who had devoted a lower total time to training during the off-season and who had a higher BMI may not be adequately prepared for the start of the season; however, this should be interpreted cautiously. First, it is important to recall that many of the injured athletes were in the “normal range” BMI category. Second, the off-season training habits were reported by the athletes and thus may be subject to recall bias. Third, the off-season training habit reports did not analyze variables associated with the off-season training programs, only total time devoted. Future investigations should prospectively evaluate alternate measures of body composition (e.g., Bod Pod) and evaluate different off-season training programs and associations with future injury.

The relationship between off-season training habits and injury is consistent with a prior study that reported a three-fold increased risk of injury in male collegiate soccer players who performed 3 h of exercise or less per week in three or more training categories [11]. The finding in this study of associations between lower levels of time devoted to training during the off-season and subsequent injury suggests that injured athletes are not physically prepared for the start of the season. In the United States, soccer players at the NCAA Division III and NAIA levels perform their off-season training programs unsupervised during the summer months. It is possible that some athletes may fail to complete all required off-season training sessions when unsupervised by the coaching staff. This preliminary evidence highlights that athletes who train less than others during the six-week period prior to the official start of the preseason are at a greater risk for injury. Coaches can use this data to inform their athletes as to the importance of off-season training not only for team success, but also as a means to help reduce their risk of some injuries. Future studies should prospectively evaluate training variables (e.g., volume and intensity) and their relationship to injury.

There are strengths and limitations to this study. Strengths associated with this study included its prospective design, the recruitment of a homogeneous sample, and the study’s sample size. Limitations associated with this study include the potential for recall bias associated with reported off-season training habits and the lack of specific details (e.g., training volume and intensity) associated with off-season training habits. In addition, the results of this study cannot be generalized to male athletes who compete at other levels of competition (e.g., professional, high school, or youth club levels).

## 5. Conclusions

Preseason SLJ and SLH scores were not associated with sports injury in female collegiate soccer players. However, a higher BMI and/or less time devoted to off-season training was associated with injury. Evaluating BMI and reviewing off-season training reports may help coaches or clinicians target at-risk athletes by modifying their training program to address potential deficits. Future research should prospectively evaluate associations between injury and alternate measures of body composition and detailed (e.g., volume and intensity) off-season training programs.

## Figures and Tables

**Table 1:** Baseline demographics, reported off-season training habits, and normalized preseason functional performance test scores (mean ± SD). (h = hours; w = week).

Training Category	Totals (n = 119)	Athletes not Injured during Season (n = 83)	Athletes Injured during Season (n = 36)	*p*-Value
Age (years)	19.2 (1.2)	19.1 (1.2)	19.4 (1.4)	0.193
Height (m)	1.65 (0.06)	1.65 (0.06)	1.65 (0.08)	0.929
Weight (kg)	62.6 (8.6)	62.2 (8.6)	63.6 (8.6)	0.405
BMI (kg/m^2^)	22.9 (2.6)	22.7 (2.7)	23.3 (2.4)	0.291
Off-Season Training Categories (h/w)				
Weightlifting (h/w)	3.1 (2.3)	3.4 (2.4)	2.4 (2.1)	0.047
Cardiovascular Exercise (h/w)	6.6 (3.3)	6.9 (3.4)	5.8 (2.6)	0.085
Plyometric Exercise (h/w)	2.9 (2.3)	3.2 (2.3)	2.1 (2.1)	0.015
Scrimmage (h/w)	3.2 (2.4)	3.2 (2.4)	3.1 (2.5)	0.777
Total Training Hours per Week	15.7 (7.9)	16.7 (8.0)	13.4 (7.2)	0.037
Functional Performance Tests (normalized to height)				
Standing Long Jump	0.80 (0.09)	0.81 (0.08)	0.80 (0.09)	0.756
(R) Single-Leg Hop	0.72 (0.09)	0.72 (0.08)	0.70 (0.10)	0.204
(L) Single-Leg Hop	0.71 (0.11)	0.71 (0.11)	0.70 (0.09)	0.371

**Table 2:** Relative risk of a noncontact time-loss lower quadrant injury based on preseason functional performance test scores.

Categories	Number per Category	All LQ Injuries N (%)	Relative Risk (95% CI)	Thigh/Knee Injuries (%)	Relative Risk (95% CI)
Standing Long Jump					
80% or more	61	20 (33)	1.0 (Reference)	12 (18)	1.0 (Reference)
79% or less	58	16 (28)	0.8 (0.5, 1.5)	8 (14)	0.7 (0.3, 1.6)
(R) Single-Leg Hop					
70% or more	72	18 (25)	1.0 (Reference)	10 (14)	1.0 (Reference)
69% or less	47	18 (38)	1.5 (0.9, 2.6)	10 (19)	1.5 (0.7, 3.4)
(L) Single-Leg Hop					
70% or more	62	16 (29)	1.0 (Reference)	10 (16)	1.0 (Reference)
69% or less	57	20 (35)	1.4 (0.8, 2.4)	10 (17)	1.1 (0.5, 2.4)
Performance on SLJ and (B) SLH					
All 3 FPT below Mean Scores ^†^	36	14 (39)	1.5 (0.9, 2.5)	7 (19)	1.2 (0.5, 2.9)
All Other Athletes	83	22 (29)	1.0 (Reference)	13 (14)	1.0 (Reference)
Limb Asymmetry between SLH					
>10 percent	24	6 (25)	0.8 (0.4, 1.7)	2 (8)	0.4 (0.1, 1.8)
10 percent or less	95	30 (32)	1.0 (Reference)	18 (19)	1.0 (Reference)
Performance on SLJ, (B) SLH, and SLH limb asymmetry					
All 3 FPT below Mean Scores ^†^ and >10% asymmetry	5	1 (20)	0.7 (0.1, 3.8)	0 (0)	Not calculated *
All Other Athletes	114	35 (31)	1.0 (Reference)	20 (18)	1.0 (Reference)

FPT = functional performance test; SLJ = standing long jump; SLH = single-leg hop; LQ = lower quadrant; ^†^ SLJ <80% height, (B) SLH <70% height; * zero injuries occurred in the “at-risk” group.

**Table 3:** Relative risk of a noncontact time-loss lower quadrant injury based on anthropometric variables and reported off-season training habits.

Categories	(N) At Risk	All LQ Injuries (%)	Relative Risk (95% CI)	Sensitivity (Sn) & Specificity (Sp) ^††^ (95% CI)	Thigh/Knee Injuries (%)	Relative Risk (95% CI)	Sensitivity (Sn) & Specificity (Sp) ^††^ (95% CI)
Body Mass Index (kg/m^2^)							
BMI ≤ 21.5	39	7 (18)	1.0 (Reference)	Sn 80.6 (64.0, 91.2)	4 (10)	1.0 (Reference)	
BMI > 21.5	80	29 (36)	2.0 (1.0, 4.2)^a^	Sp 38.6 (28.1, 49.9)	16 (20)	2.0 (0.7, 5.4)	
Weightlifting (hr/wk)							
4 hr or more per week	30	9 (20)	1.0 (Reference)		6 (13)	1.0 (Reference)	
<4 hr per week	89	27 (37)	1.8 (0.9, 3.5)		14 (19)	1.4 (0.6, 3.4)	
Cardiovascular Exercise							
5 hr or more per week	36	15 (24)	1.0 (Reference)		11 (19)	1.0 (Reference)	
<5 hr per week	83	21 (50)	1.3 (0.8, 2.2)		9 (15)	0.8 (0.3, 1.7)	
Plyometric Exercise							
3 hr or more per week	51	9 (18)	1.0 (Reference)	Sn 75.0 (57.8, 87.9)	6 (12)	1.0 (Reference)	
<3 hr per week	68	27 (40)	2.3 (1.2, 4.4)^b^	Sp 50.6 (39.4, 61.8)	14 (21)	1.8 (0.7, 4.2)	
Scrimmaging							
3.2 hr or more per week	49	14 (29)	1.0 (Reference)		9 (18)	1.0 (Reference)	
<3.2 hr per week	70	22 (31)	1.1 (0.6, 1.9)		11 (16)	0.9 (0.4, 1.9)	
Total Time Training per Week							
14.75 hr or more per week	60	10 (17)	1.0 (Reference)	Sn 72.2 (54.8, 85.8)	6 (10)	1.0 (Reference)	Sn 70.0 (45.7, 88.1)
<14.75 hr per week	59	26 (47)	2.6 (1.4, 5.0)^c^	Sp 60.2 (48.9, 70.8)	14 (24)	2.4 (1.0, 5.8)^d^	Sp 54.6 (44.2, 64.6)
High BMI and Low Total Training Time per Week							
BMI > 21.5 & <14.75 hr/wk	47	24 (51)	3.1 (1.7, 5.5)^e^	Sn 66.7 (49.0, 81.4)	13 (28)	2.8 (1.2, 6.6)^f^	Sn 65.0 (40.8, 84.6)
All Other Athletes	72	12 (17)	1.0 (Reference)	Sp 72.3 (61.4, 81.6)	7 (10)	1.0 (Reference)	Sp 67.0 (57.0, 75.9)
FPT Performance and Total Time Training							
Suboptimal performance on 3 FPT & < 14.75 hr per week training	12	8 (47)	1.7 (0.9, 3.1)		3 (18)	1.1 (0.3, 3.2)	
All Other Athletes	107	28 (29)	1.0 (Reference)		17 (17)	1.0 (Reference)	
High BMI, Low Total Training Time per Week, and Suboptimal FPT Performance							
Suboptimal FPT Performance, BMI > 21.5, Total Training Time per Week < 14.75 hr/week	12	7 (58)	2.2 (1.2, 3.8)^g^	Sn 19.4 (8.2, 36.0)	3 (25)	1.6 (0.5, 4.6)	
All Other Athletes	107	29 (27)	1.0 (Reference)	Sp 86.7 (77.9, 92.9)	17 (16)	1.0 (Reference)	

Suboptimal FPT Performance = SLJ < 80% height, (B) SLH < 70% height; FPT = functional performance test; ^††^ sensitivity (Sn) and specificity (Sp) values provided for significant associations; ^a^ *p*-value = 0.04; ^b^ *p*-value = 0.01; ^c^ *p*-value = 0.001; ^d^ *p*-value = 0.05; ^e^ *p*-value = 0.0001; ^f^ *p*-value = 0.014; ^g^ *p*-value = 0.04.
